# The predictive value of necroptosis-related lncRNAs in prostate cancer prognosis and their potential to distinguish between cold and hot tumors

**DOI:** 10.3389/fcell.2025.1722671

**Published:** 2026-01-05

**Authors:** Ke Zhang, Kai Li, Junpeng Deng, Chunchun Zhao, Fei Wang, JianJun Xie

**Affiliations:** Department of Urology, The Affiliated Suzhou Hospital of Nanjing Medical University, Suzhou Municipal Hospital, Gusu School, Nanjing Medical University, Suzhou, China

**Keywords:** cold-hot tumors, immune profiling, immunotherapy, necroptosis, prostate adenocarcinoma

## Abstract

**Background:**

Prostate cancer (PCa) is frequently associated with poor prognosis, and immunotherapy has shown limited efficacy. This study aimed to identify novel necroptosis-related long non-coding RNAs (lncRNAs) that could predict patient outcomes and guide personalized treatment.

**Methods:**

Transcriptomic data from The Cancer Genome Atlas (TCGA) were analyzed using co-expression analysis and univariate Cox regression to identify lncRNAs associated with PCa progression. A necroptosis-related lncRNA prognostic model was constructed using Least Absolute Shrinkage and Selection Operator (LASSO) and validated via Kaplan-Meier survival analysis, time-dependent receiver operating characteristic (ROC) curves, Cox regression, and calibration plots. Functional analyses included Gene set enrichment analysis (GSEA), principal component analysis (PCA), immune profiling, and half-maximal inhibitory concentration (IC50) predictions to explore therapeutic implications.

**Results:**

We established a nine-lncRNA necroptosis-related signature with strong prognostic performance. Among these, NR2F1-AS1 was identified as a core oncogenic lncRNA, showing marked upregulation in PCa tissues and promoting proliferation, invasion, and migration *in vitro*. The two inferred risk groups demonstrated distinct immune characteristics: hot tumors (Cluster 2) exhibited higher infiltration of activated immune cells, increased immune checkpoint expression, and greater predicted sensitivity to immunotherapy, whereas cold tumors showed immunosuppressive infiltration patterns and lower checkpoint levels. These features allowed the model to robustly distinguish cold from hot tumor phenotypes.

**Conclusion:**

Necroptosis-related lncRNAs, particularly NR2F1-AS1, may serve as prognostic biomarkers and inform immune-based stratification, supporting more precise personalized treatment strategies for PCa.

## 1 Introduction

Prostate cancer is a malignant epithelial tumor of the prostate and one of the most common cancers of the male genitourinary system. According to global cancer statistics, PCa accounted for 14.1% of all newly diagnosed cancers and 6.8% of cancer-related deaths among men in 2020 (Sung et al., 2021). Although the incidence of PCa in China is relatively low, it has been rising steadily due to population aging, lifestyle changes, and improvements in diagnostic strategies, making it an increasingly significant threat to male health (Chen et al., 2016). Due to substantial tumor heterogeneity, personalized and precision therapy has become a central focus in PCa management. Current therapeutic strategies include active surveillance, radical prostatectomy or radiotherapy for localized disease and hormonal deprivation therapy and other systemic therapies in metastatic cases (Mattes, 2024; Azad et al., 2025).

Nonetheless, given all standard care, progression to castration-resistant prostate cancer (CRPC) is inevitable is some cases and the tumoral resistance to apoptosis necessitated other pathways to be investigated as a potential therapeutic approach (Tang et al., 2020). Unlike apoptosis, necroptosis is a programmed inflammatory form of cell death that can activate the tumor microenvironment and enhance CD8^+^ T cell–mediated antitumor immunity (Galon and Bruni, 2019). For instance, necroptotic tumor-mimicking nanoparticle vaccines have been shown to expand CD8^+^ T cells and potentiate antitumor responses in mouse models (Snyder et al., 2019). In gastric cancer, researchers have identified that necroptotic pyroptosis can generate an immune-suppressive tumor microenvironment (TME), promoting the malignant progression of gastric cancer and suggesting it as a potential immunotherapeutic target (Gong et al., 2019). However, the role and underlying mechanisms of necroptosis in PCa remain poorly understood.

LncRNAs regulate gene expression through interactions with proteins and other RNA molecules (Quinn and Chang, 2016). In hepatocellular carcinoma, lncRNAs have been shown to induce necroptosis by modulating microRNA-targeted mRNAs, while also protecting cancer cells from external stress via inhibition of the NF-κB necroptotic signaling pathway (Dragomir et al., 2020; Jiang et al., 2021). Additionally, lncRNAs may promote tumor-associated inflammation, facilitating immune evasion and disease progression. Despite these insights, the roles of necroptosis-related lncRNAs in PCa remain largely unexplored. Given their potential to modulate necroptotic pathways and the tumor microenvironment, necroptosis-related lncRNAs may influence immune cell infiltration and tumor immunogenicity, thereby associating with cold and hot tumor phenotypes.

Differentiating between cold and hot tumors is crucial for improving immunotherapy efficacy, as converting cold tumors into hot tumors can substantially enhance treatment responses. However, effective tumor classification methods remain limited in clinical practice. Recently, circulating lncRNAs have emerged as promising cancer biomarkers. Therefore, this study aims to reclassify PCa patients based on necroptosis-related lncRNAs, enabling the identification of hot tumors and facilitating more precise prognostic assessment and personalized therapeutic strategies (Yuan et al., 2020).

## 2 Materials and methods

### 2.1 Retrieval of basic information of PCa patients from public databases

We obtained expression matrices of prostate adenocarcinoma and normal tissues from TCGA (TCGA-PRAD, https://portal.gdc.cancer.gov/). The mRNA transcriptome data and relevant clinical information were downloaded, and the data were converted from FPKM to TPM format. To mitigate statistical biases, we excluded patients with missing overall survival (OS) values or OS less than 30 days. In total, 588 patients were included. Using Strawberry Perl and the R package “caret” (version 6.0–93) (Yavorska and Burgess, 2017), these patients were randomly allocated into train and test risk groups with an equal ratio.

### 2.2 Selection of necroptosis-related genes and LncRNAs

We downloaded the necroptosis-related necroptosis gene set M24779.GMT, containing 8 genes, from GSEA (http://www.gsea-msigdb.org/gsea/index.jsp). Based on previously reported information on necroptosis, we curated a map of 67 necroptosis-related genes (Raza et al., 2004). Using Strawberry Perl and the “limma” package (Chen et al., 2022b), we screened for differentially expressed genes and identified a total of 5022 differentially expressed lncRNAs, applying criteria of Log2 fold change (FC) > 1, false discovery rate (FDR) < 0.05, and p < 0.05 (Chen et al., 2022a). Subsequently, we performed a correlation analysis between the 67 necroptosis-related genes and the differentially expressed lncRNAs, identifying 387 lncRNAs significantly correlated with necroptosis-related apoptosis. Criteria for selecting necroptosis-related lncRNAs included a Pearson correlation coefficient >0.4 and P < 0.001.

### 2.3 Establishment of risk model and independent prognostic analysis

Clinical information of patients was retrieved from the TCGA database, and necroptosis-related lncRNAs were selected through univariate and multivariate Cox regression analyses. A prognostic model was then constructed using LASSO regression with 10-fold cross-validation, 1000 iterations, and a significance threshold of P < 0.05. The risk score for each patient was calculated using the following formula:

$$\text{risk score}=\sum_{k=1}^{n}\text{coef}\left({\text{lncRNA}}^{k}\right)*\text{expr}\left({\text{lncRNA}}^{k}\right),$$

where coef (lncRNA_n) represents the coefficient of the lncRNA associated with survival, and expr (lncRNA_n) represents the expression level of the lncRNA. Patients were categorized into low- and high-risk groups based on the median risk score (Chen et al., 2024). Time-dependent ROC curves at 1, 2, and 3 years were used to evaluate the prognostic performance of the model. Finally, univariate and multivariate Cox regression analyses were performed to determine whether the risk score served as an independent prognostic factor beyond clinicopathological variables, and ROC analysis was used to compare the prognostic value of the risk score with traditional pathological features.

### 2.4 Forest plot analysis

We constructed nomograms for 1-year, 2-year, and 3-year OS based on age, tumor stage, and risk score. The forest plots were generated using the “rms” package (Shao et al., 2023) to facilitate multi-index joint diagnosis or prediction of disease risk or prognosis. Nomograms provide a precise, digital probability of survival or risk for each patient.

### 2.5 Enrichment analysis

We utilized the KEGG gene set (kegg.v7.4.symbols.gmt) and GSEA software (https://www.gsea-msigdb.org/gsea/login.jsp) to distinguish significantly enriched pathways between low and high-risk groups. Pathways were considered significantly enriched based on a cutoff of P < 0.05 and false discovery rate (FDR) < 0.25.

### 2.6 Exploration of TME and immune checkpoints

Based on the GSEA analysis results mentioned above, we further explored the immune factors of patients. Initially, we assessed the immune cell infiltration status of PCa patients in the TCGA database using TIMER2.0 (http://timer.cistrome.org/). Additionally, infiltration data of TCGA-PRAD tumors were downloaded, and differences in immune cell infiltration were analyzed using the Mann-Whitney U test, limma (version 3.52.2), ggplot2 (version 3.3.6), and other R packages (Hashemi Sheikhshabani et al., 2023). Furthermore, we utilized the “ggpubr” package (Chen et al., 2021) to explore TME scores and immune checkpoint status between low and high-risk groups.

### 2.7 Application of disease models in clinical drug therapy

We evaluated the sensitivity analysis of each PCa patient in the CancerRxGene (GDSC) database (https://www.cancerrxgene.org/) to determine their treatment response based on IC50. The “pRRophetic” R package was instrumental in this analysis.

### 2.8 Clustering analysis of 9 independent prognostic LncRNAs

To explore the response of PCa to immune therapy, we performed clustering analysis using the “ConsensusClusterPlus” (Version 0.5) R package (Li et al., 2021a) to identify potential molecular subgroups based on the expression profiles of prognostic lncRNAs. PCA, t-distributed Stochastic Neighbor Embedding (t-SNE), and Kaplan-Meier survival analysis were conducted using the “Rtsne” package (version 0.15) (Li et al., 2022). Additionally, we utilized the “pRRophetic” package (Wang et al., 2024) to compare drug sensitivity analysis.

### 2.9 Cell culture and transfection

PCa cell lines (LNCap and PC3) were obtained from the Cell Bank of the Chinese Academy of Sciences (Beijing, China). Both cell lines were cultured in DMEM medium supplemented with 10% fetal bovine serum (FBS) and 2% penicillin-streptomycin in a humidified incubator with 5% CO_2_ at 37 °C. Transfections of siRNA and overexpression plasmids were performed using Lipofectamine 2000 (Invitrogen, Carlsbad, California, United States) following the manufacturer’s instructions in LNCap and PC3 cells.

### 2.10 Real-time quantitative reverse transcription polymerase chain reaction (qRT-PCR)

To assess the expression levels of NR2F1-AS1 mRNA in cells and evaluate the efficiency of siRNA and overexpression transfections, total RNA was extracted from harvested cells using a PCR extraction kit (Cusabio, Shanghai, China, CAT: 19231ES50) according to the manufacturer’s instructions. PCR primers were purchased from Sengen Biotech Co., Ltd. Primer sequences are listed in Table 1.

**TABLE 1:** Primer sequences.

Amplicons	Sequences
NR2F1-AS1	Forward primer: CATGCCGTGATGTAAGCTGC Reverse primer: TCTGTCCCAGTCCTAGAGGC
GAPDH	Forward primer: GAATGGGCAGCCGTTAGGAA Reverse primer: GAGGGATCTCGCTCCTGGAA

### 2.11 Cell counting Kit-8 (CCK-8), clonogenic, and scratch assays

Cell proliferation assay: LNCap and PC3 cells were seeded in 96-well plates at a density of 2 × 10^2^ cells per well under different lipid transfection conditions and cultured at 37 °C with 5% CO_2_. At 24, 48, 72, and 96 h, 10 μL of CCK-8 solution (Dojindo Molecular Technologies, Inc., Kumamoto, Japan) was added to each well, and absorbance was measured at 450 nm to plot cell growth curves and analyze the data.

Clonogenic assay: LNCap and PC3 cells were seeded in 6-well plates at a density of 10^3^ cells per well under different treatment conditions and cultured at 37 °C with 5% CO_2_ for 10–14 days. Cells were then fixed with 4% paraformaldehyde, stained with crystal violet, and observed for colony formation under a microscope.

Transwell assay: LNCap and PC3 cells at a density of 2 × 10^4^cells/mL were seeded into the upper chamber containing serum-free medium under different treatment conditions. The lower chamber contained DMEM supplemented with 10% FBS as a chemoattractant. After 48 h of incubation at 37 °C with 5% CO_2_, non-migrated cells on the upper surface were removed. Cells that had migrated or invaded to the lower surface were fixed with 4% paraformaldehyde, stained with crystal violet, and manually counted for analysis.

Scratch assay: Cells under different treatment conditions were seeded at a density of 2 × 10^6^ cells per well in 6-well plates until reaching 80%–90% confluence. A scratch was made swiftly with the tip of a 10 μL pipette tip. After washing twice with PBS to remove cell debris, the plate was incubated in a serum-free medium. Scratch closure was observed using fluorescence microscopy at 0, 24, and 48 h after scratching.

### 2.12 Statistical analysis

All statistical analyses were conducted using R software (version 4.2.1). Immune infiltration analyses and comparisons between groups were assessed using the Mann-Whitney U test. For cell-based assays, statistical significance was determined using one-way ANOVA. A two-sided P < 0.05 was considered statistically significant.

## 3 Results

### 3.1 Identification of necroptosis-related LncRNAs in PCa patients

**FIGURE 1:**
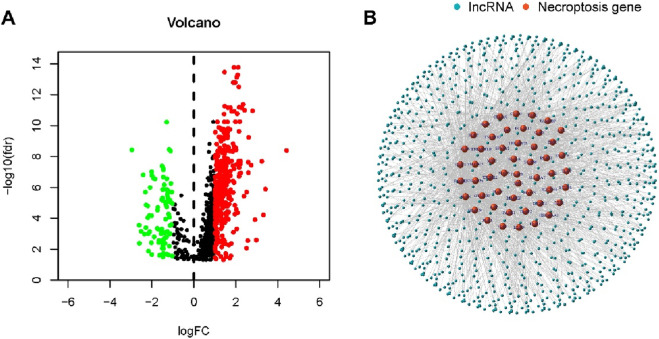
Identification of necroptosis-related lncRNAs in patients with PCa. **(A)** Volcano plot showing differentially expressed necroptosis-related genes between PCa and normal prostate tissues. **(B)** Co-expression network of necroptosis-related genes and long non-coding RNAs (lncRNAs), illustrating interactions with correlation coefficient >0.5 and P < 0.001.

We obtained 302 tumor samples and 40 normal samples from the TCGA dataset. Differential expression analysis between normal and tumor samples revealed 387 necroptosis-associated lncRNAs based on the expression of 67 necroptosis-related genes (|Log2FC| > 1, P < 0.05). Among these, 194 were upregulated and 193 were downregulated (Figure 1A). Figure 1B illustrates the network interaction between necroptosis-related genes and lncRNAs.

### 3.2 Construction and validation of necroptosis-related LncRNA model in PCa

**FIGURE 2:**
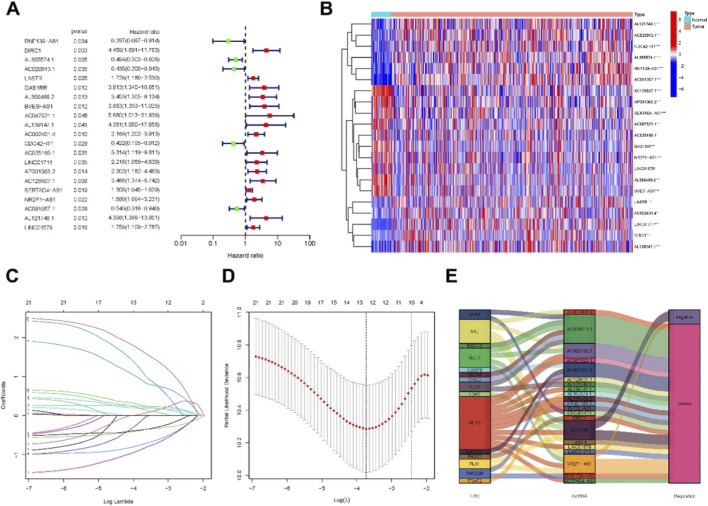
Extraction of necroptosis-related lncRNAs prognostic signature in PCa. **(A)** Univariate Cox regression identified necroptosis-related lncRNAs associated with overall survival in patients with PCa. **(B)** Expression heatmap showing the profiles of the 30 prognostic necroptosis-related lncRNAs. **(C)** Ten-fold cross-validation for variable selection using the LASSO regression model. **(D)** LASSO coefficient profiles showing the selection of 9 necroptosis-related lncRNAs that constituted the prognostic signature. **(E)** Co-expression network diagram of necroptosis-related genes and corresponding lncRNAs.

Using univariate Cox regression analysis, we identified 9 necroptosis-related lncRNAs significantly correlated with OS in PCa patients, as illustrated in a forest plot (Figure 2A) and heatmap (Figure 2B). To reduce overfitting, these lncRNAs were further validated using Lasso regression analysis (Figures 2C,D). A Sankey diagram was used to visualize the 9 necroptosis-related lncRNAs and their associations with patient prognosis (Figure 2E), highlighting which lncRNAs contributed to higher or lower risk.

**FIGURE 3:**
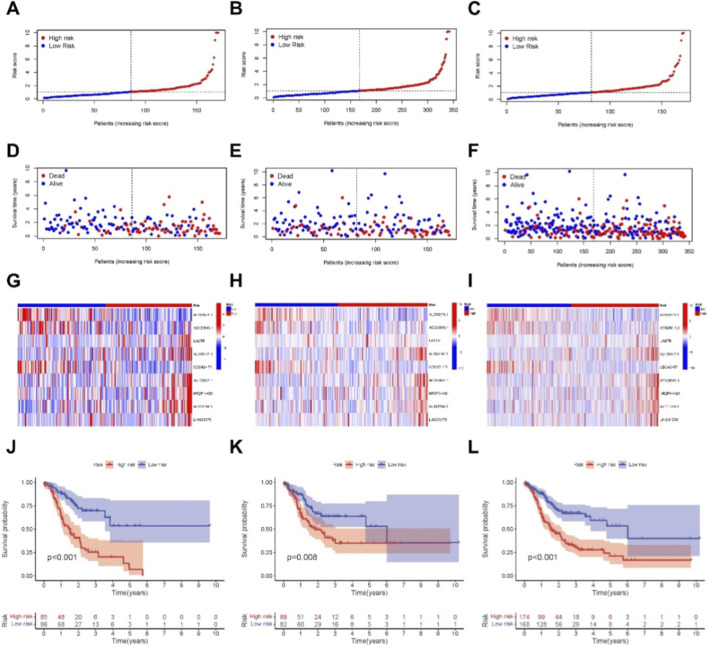
Prognosis value of the 9necroptosis-related lncRNAs model in the train, test, and all sets. **(A–C)** Distribution of risk scores based on the necroptosis-related lncRNA signature in the training, testing, and entire cohorts, respectively. **(D–F)** Overall survival time and status for patients in low- and high-risk groups in the training, testing, and entire cohorts, respectively. **(G–I)** Heatmaps showing the expression patterns of the 9 necroptosis-related lncRNAs in the three cohorts. **(J–L)** Kaplan-Meier survival curves comparing overall survival (OS) between the low- and high-risk subgroups in the training, testing, and entire cohorts.

Based on patient characteristics, we computed a risk score for each patient using the formula: Risk Score = AL355574.1 × (−0.6096) + AC020913.1 × (−0.9605) + LASTR × (−0.3135) + AL139147.1 × (2.05953) + CDC42-IT1 × (1.3234) + AC129507.1 × (1.8770) + NR2F1-AS1 × (0.9888) + AL121748.1 × (2.3694) + LINC01579 × (0.6252). Patients in the training set, testing set, and entire cohort were stratified into high- and low-risk groups based on the median risk score. After adjusting the risk values for clarity (Figures 3A–C), higher mortality rates were observed in the high-risk group compared to the low-risk group within the TCGA-PRAD dataset (Figures 3D–F). Heatmap visualizations further depicted differential expression patterns of model-associated lncRNAs between high- and low-risk groups across datasets (Figures 3G–I). Kaplan–Meier survival analysis demonstrated significantly lower OS in the high-risk group, confirming the prognostic utility of the necroptosis-related ncRNA model (Figures 3J–L).

### 3.3 Construction and evaluation of nomogram and ROC curve

**FIGURE 4:**
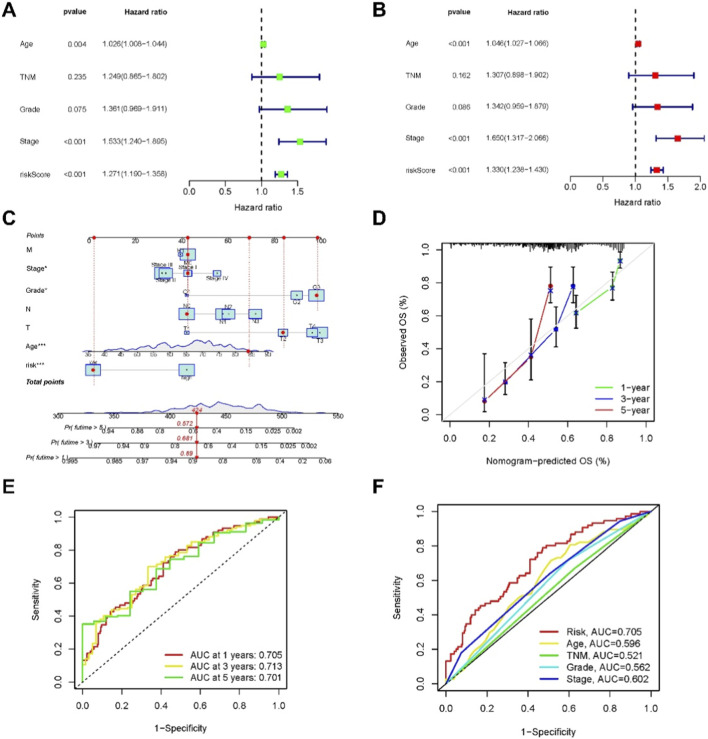
Nomogram and assessment of the risk model. **(A,B)** Univariate and multivariate Cox regression analyses of clinical factors and risk score with OS. **(C)** Nomogram integrating risk score, age, and tumor stage for predicting 1-, 2-, and 3-year OS. **(D)** Calibration curves for 1-, 2-, and 3-year OS based on the nomogram. **(E)** ROC curves at 1, 2, and 3 years in the training, testing, and entire cohorts. **(F)** Comparison of 3-year ROC curves among the risk score, nomogram total score, and clinical parameters. We refer to TNM stage as pathological stage, whereas “stage” denotes clinical stage.

In univariate Cox regression analysis, the hazard ratio (HR) and 95% confidence interval (CI) for the risk score were 1.27 (1.1899–1.3580, P < 0.001). For Age, the HR was 1.02 (1.0082–1.0438, P < 0.005), and for Stage, the HR was 1.53 (1.2404–1.8953, P < 0.001) (Figure 4A). In multivariate Cox regression analysis, the HR for the risk score was 1.33 (1.2377–1.4302, P < 0.001), for Age 1.05 (1.0266–1.0660, P < 0.001), and for Stage 1.65 (1.3170–2.0664, P < 0.001) (Figure 4B). These three independent prognostic factors were integrated to construct a nomogram predicting 1-year, 2-year, and 3-year OS in PCa patients (Figure 4C).

Calibration plots indicated good agreement between predicted and observed OS rates at 1, 2, and 3 years (Figure 4D). ROC curve analysis demonstrated the predictive performance of the nomogram, with AUC values of 0.705, 0.713, and 0.701 for 1-year, 3-year, and 5-year OS, respectively (Figure 4E). In comparison, conventional clinical factors including Age, TNM (pathological stage), Grade, and Stage (clinical stage) showed lower predictive accuracy, with AUC values of 0.596, 0.521, 0.562, and 0.602, respectively (Figure 4F). These results indicate that the necroptosis-related lncRNA risk score provides robust prognostic value beyond traditional clinical parameters.

### 3.4 GSEA enrichment analysis

**FIGURE 5:**
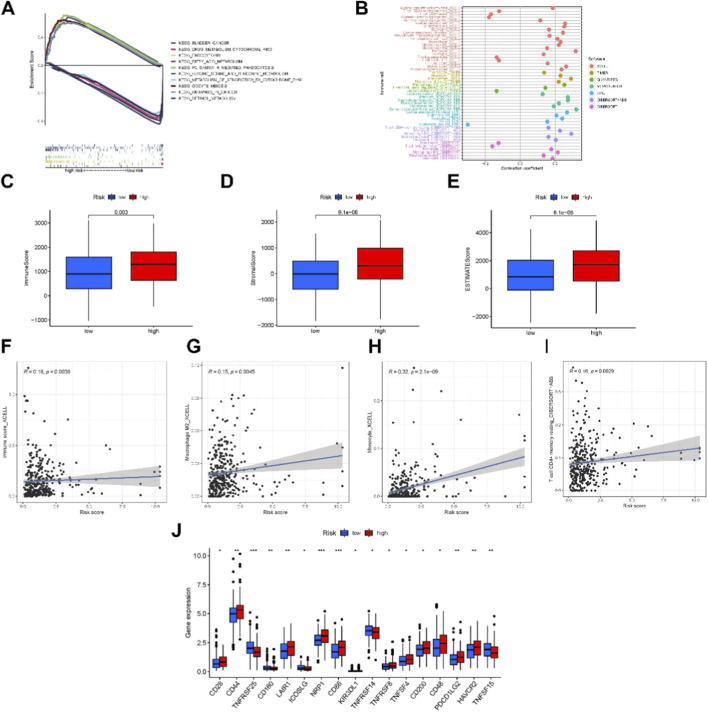
The investigation of tumor immune factors and immunotherapy. **(A)** GSEA showing the top 10 pathways significantly enriched in the high-risk group. **(B)** Bubble plot depicting the distribution of immune cells across risk groups. **(C–E)** Correlation analysis between risk score and infiltration levels of specific immune cell types. **(F–I)** Comparison of multiple immune-related scores between high- and low-risk groups. **(J)** Differential expression of 15 immune checkpoints between risk groups, highlighting potential immunotherapy targets.

To investigate biological functional differences between high- and low-risk groups, we conducted a GSEA comparing KEGG pathways in the entire cohort. The top 5 enriched pathways for each group were selected. The high-risk group was prominently associated with pathways related to tumor invasion, drug metabolism, and lipid metabolism, whereas the low-risk group was mainly associated with carbohydrate metabolism pathways (Figure 5A). Notably, the enrichment of drug metabolism pathways in the high-risk group may reflect altered xenobiotic processing and chemotherapeutic response, suggesting potential implications for treatment resistance in PCa.

We further investigated the immune landscape and clinical treatment correlations within the high-risk group using immune cell bubble plots. Significant associations (P < 0.05) were observed between the high-risk group and multiple immune cell types, including Myeloid dendritic cells (activated, XCELL), CD4^+^ central memory T cells (XCELL), naive CD8^+^ T cells (XCELL), M2 macrophages (QUANTISEQ), non-regulatory CD4^+^ T cells (QUANTISEQ), uncharacterized cells (QUANTISEC), CD8^+^ T cells (TIMER), neutrophils (TIMER), cancer-associated fibroblasts (EPIC), endothelial cells (EPIC), macrophages (EPIC), naive B cells (CIBERSORT), and follicular helper T cells (CIBERSORT) (Figure 5B).

Additionally, higher risk scores were significantly correlated with M2 macrophages, monocytes, and resting CD4^+^ memory T cells, which are immune cells that play critical roles in PCa immunotherapy (Figures 5C–E). Compared with the low-risk group, the high-risk group exhibited higher immune and microenvironment scores, indicating pronounced differences in immune infiltration and TME status (Figures 5F–I). Importantly, multiple immune checkpoints showed stronger activation in the high-risk group (Figure 5J), suggesting that patients in this group may benefit more from checkpoint inhibitor therapies.

Collectively, these findings indicate that our necroptosis-related lncRNA risk model not only reflects differences in tumor biology and immune infiltration but may also guide the selection of personalized immunotherapy strategies for PCa patients.

### 3.5 Analysis of PCa tumor immune subtypes

**FIGURE 6:**
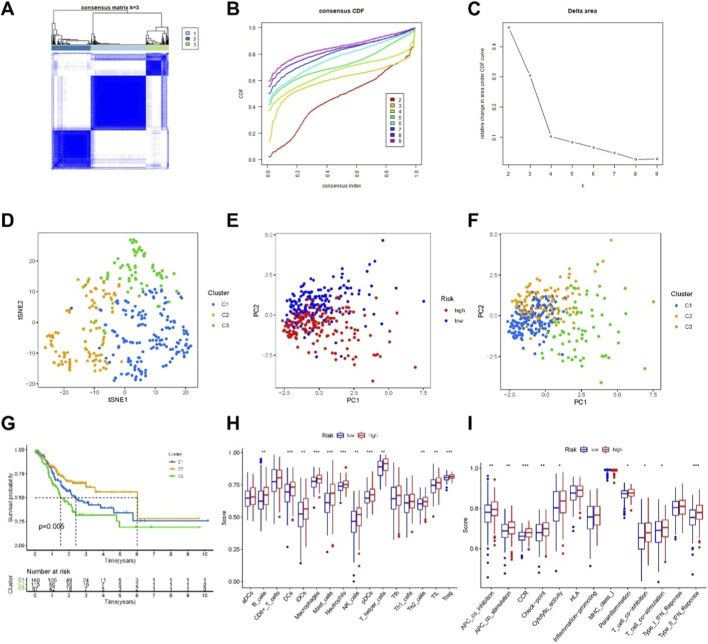
Distinction between cold and hot tumors and immunotherapy prediction. **(A–C)** Patients are divided into two clusters by ConsensusClusterPlus. **(D–F)** The t-SNE of two clusters. **(G)** Kaplan–Meier survival curves of OS in clusters. **(H–I)** The ssGSEA scores of immune cells and immune functions in Clusters.

Different clusters or subtypes in tumor research often exhibit distinct changes in the tumor immune microenvironment, leading to varied responses to immune therapy [21,22]. In this study, we identified 9 necroptosis-related lncRNAs and analyzed RNA sequencing data from TCGA-PRAD, focusing on 67 necroptosis genes and 342 associated lncRNAs. We set the criteria as correlation coefficient >0.4 and P < 0.001 and used the “ConsensusClusterPlus” package to classify PCa patients into two groups. We found that t-distributed stochastic neighbor embedding (tSNE) clearly distinguished three clusters (Figures 6A–C). Furthermore, we validated distinct PCA distributions among the two risk groups and three different clusters (Figures 6D–F). Kaplan-Meier analysis further revealed that Cluster 2 exhibited better OS (P = 0.005) (Figure 6G). We also showed the single-sample gene set enrichment analysis (ssGSEA) scores of immune cells and immune functions in Clusters in Figures 6H,I.

**FIGURE 7:**
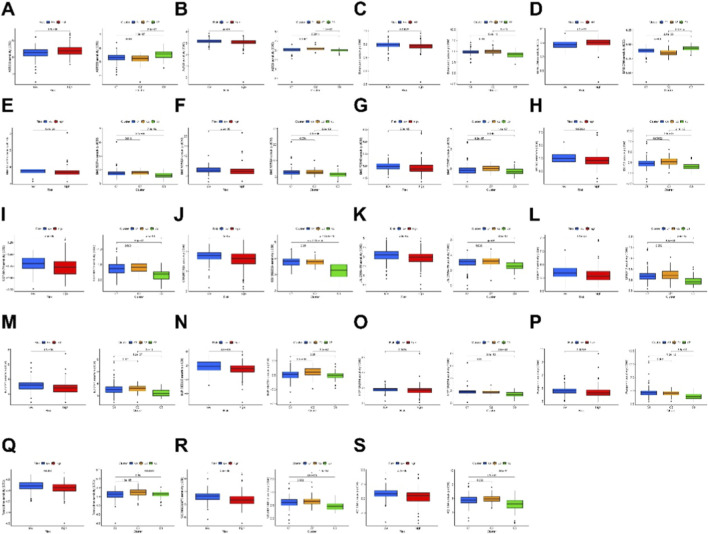
Immunotherapeutic drugs showing significant differences in IC50 values. **(A–S)** Boxplots showing the predicted IC50 values of 19 immunotherapeutic drugs between the low-risk and high-risk groups (left panels) and among different molecular clusters (right panels). Statistical significance was assessed between groups as indicated.

Finally, our comparative analysis of drug sensitivity revealed significant differences in the IC50 values of 19 immune therapy drugs such as Bexarotene, Pazopanib, Roscovitine, and GSK269962A across different clusters and risk groups, highlighting potential variations in response to 19 chemotherapeutic or targeted drugs used for systemic treatment of PCa (Figures 7A–S). In summary, based on these analyses, we propose further investigation into personalized immune therapy responses in PCa patients and exploration of principles for precision medicine, laying a solid foundation for personalized treatment strategies.

### 3.6 Inhibition of NR2F1-AS1 can suppress the proliferation, invasion, and migration of PCa cells

**FIGURE 8:**
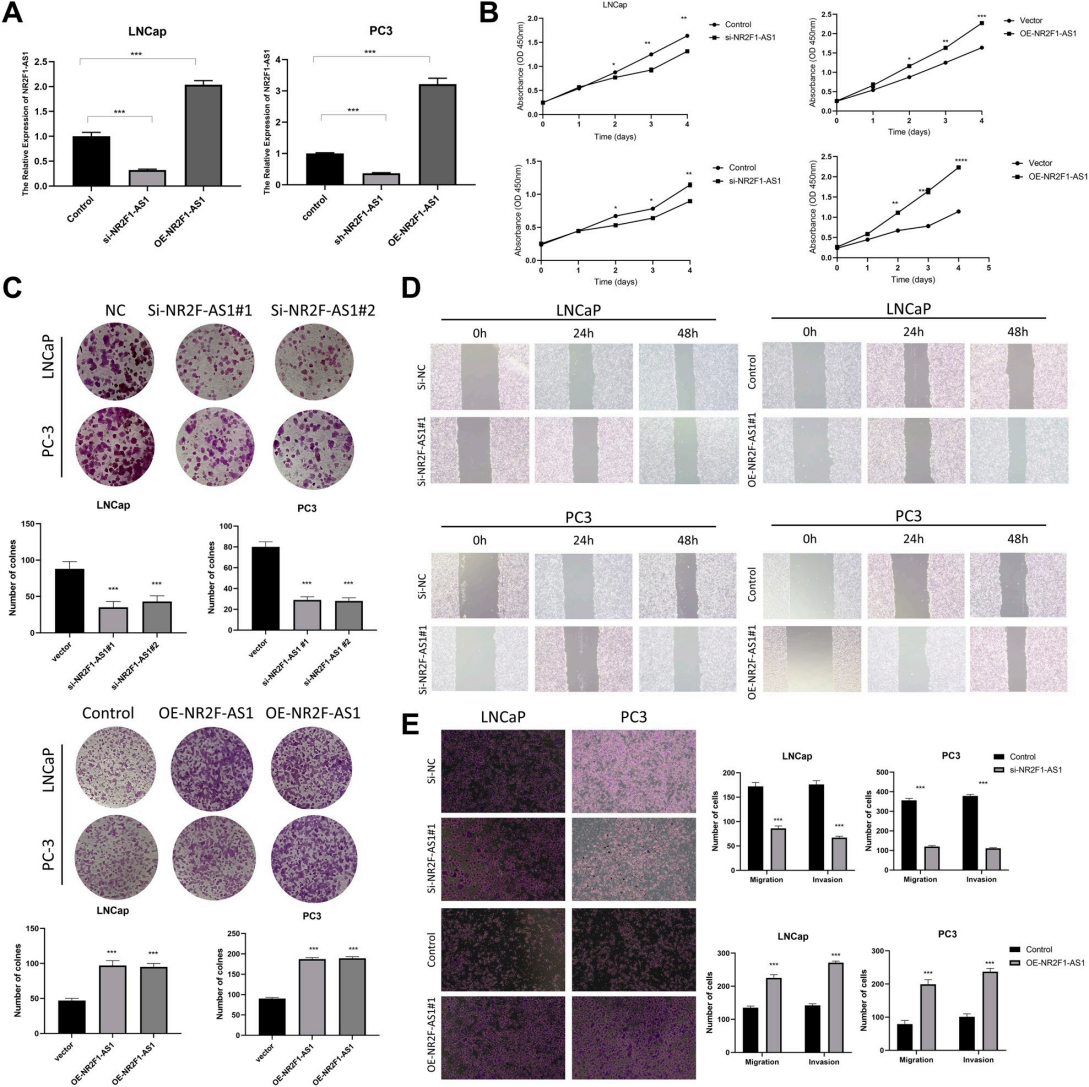
*In vitro* validation of NR2F1-AS1 overexpression promoting proliferation, invasion, and migration of PCa cells. **(A)** qRT-PCR assessment of knockdown and overexpression efficiency of NR2F1-AS1 in two cell lines (N = 3). **(B,C)** CCK-8 and colony formation assays confirm the effect of NR2F1-AS1 on PCa cell proliferation (N = 3). **(D,E)** Scratch wound healing and transwell assays evaluating the impact of NR2F1-AS1 on PCa cell migration and invasion (N = 3). Significance in **(A–E)** was determined by one-way ANOVA. *P < 0.05, **P < 0.01, ***P < 0.001. Representative images are shown, and all experiments were independently repeated three times. Scale bar = 100 μm.

Based on the above research findings, NR2F1-AS1 was hypothesized to have oncogenic effects in PCa. To validate this, we performed a series of *in vitro* experiments. Knockdown and overexpression efficiencies of NR2F1-AS1 were confirmed in LNCap and PC3 cell lines (Figure 8A). CCK-8 assays demonstrated that silencing NR2F1-AS1 significantly inhibited cell proliferation, whereas overexpression promoted proliferation (Figure 8B). Consistently, colony formation and wound healing assays showed reduced clonogenicity and migration upon NR2F1-AS1 knockdown, while overexpression enhanced these phenotypes (Figures 8C,D). Transwell migration and invasion assays further confirmed that NR2F1-AS1 promotes metastatic capabilities in PCa cells (Figure 8E).

Importantly, in the context of immune stratification, Cluster 2, which corresponds to hot tumors with higher immune cell infiltration and elevated immune checkpoint expression, exhibited better OS (P = 0.005). These findings suggest that NR2F1-AS1 may influence both tumor aggressiveness and the immune microenvironment, highlighting its potential as a therapeutic target and as a biomarker for guiding immunotherapy in PCa patients.

## 4 Discussion

PCa remains a clinical challenge due to its heterogeneity and limited response to immunotherapy. Classified as a “cold” tumor, PCa exhibits low T-cell infiltration and an immunosuppressive TME, resulting in limited efficacy of current immune checkpoint inhibitors (ICIs), such as CTLA-4 and PD-1/PD-L1 blockade in mCRPC (Sena et al., 2021; Tsaur et al., 2021; Roberts et al., 2022). These challenges highlight the need for novel biomarkers and combination strategies to enhance immune responses and guide patient stratification (Handa et al., 2020; Subudhi et al., 2021).

Existing prognostic models for PCa, based on lncRNAs or mRNAs, provide limited insight into immune stratification. To address these limitations, we developed a 9 necroptosis-related lncRNA model that predicts patient prognosis and distinguishes cold and hot tumors. Using this model, patients were stratified into high- and low-risk groups, showing significant differences in survival outcomes and predicted sensitivity to systemic therapies. Molecular clustering further revealed distinct immune microenvironments: Cluster 2 corresponded to hot tumors, characterized by higher immune cell infiltration, elevated checkpoint expression, and improved overall survival. These results suggest that our model offers improved prognostic accuracy and immune-typing specificity, providing a valuable tool for personalized immunotherapy in PCa.

Among the 9 lncRNAs, NR2F1-AS1 emerged as a key lncRNA with higher expression in PCa tissues. While its role in PCa has been underexplored, NR2F1-AS1 has been reported to regulate proliferation, invasion, migration, apoptosis, and metabolic pathways in various cancers, including HCC, gastric, colorectal, pancreatic, esophageal, breast, lung, and thyroid cancers (Huang et al., 2018; Tarchini et al., 2018; Guo et al., 2019; Zhang et al., 2019; Sanchez Calle et al., 2020; Wang et al., 2020; Yang et al., 2020; Zhang C. et al., 2020; Zhang Q. et al., 2020; Li et al., 2021b; Liao et al., 2021; Luo et al., 2021; Lv et al., 2021). Importantly, necroptosis and lncRNAs both regulate cell death, though through distinct mechanisms (Wei et al., 2020). NR2F1-AS1 may interact with necroptosis pathways to influence tumor cell survival and modulate the immune microenvironment, potentially affecting sensitivity to immunotherapy. Our *in vitro* experiments confirmed that silencing NR2F1-AS1 inhibits proliferation, migration, and invasion of PCa cells, indicating its oncogenic function.

For comparison with previous studies, several lncRNA-based prognostic models have been developed in PCa, including a 7-lncRNA signature (Ledesma-Bazan et al., 2024) and a 4-lncRNA signature (Mulati et al., 2024). However, these models primarily focused on biochemical recurrence-free survival (BRFS) rather than OS. In addition, currently available genomic prediction platforms for PCa such as the Oncotype Dx Genomic Prostate Score® and the Decipher® genomic-clinical classifier generally report AUCs around 0.63 and 0.69, respectively. In comparison, our 9-necroptosis-related lncRNA model showed superior predictive accuracy for OS (AUC = 0.705) and maintained discrimination across multiple clinical subgroups. Notably, whereas most existing models were constructed without considering regulated cell death pathways, our model integrates necroptosis-related signatures, thereby providing mechanistic relevance and improving potential clinical applicability.

Several limitations of this study should be acknowledged. First, our analyses were based on retrospective TCGA data. External validation was not available in the present study due to lack of compatible datasets, and therefore further validation in independent cohorts such as GEO prostate cancer datasets will be required in the future. Second, prostate-specific antigen (PSA) information was not available in the TCGA dataset, and therefore could not be incorporated into the multivariate analysis and nomogram construction. This represents an important limitation and should be addressed in future studies using datasets with complete clinical variables. Third, although NR2F1-AS1 was experimentally validated as an oncogenic lncRNA in PCa, the detailed downstream regulatory mechanisms and its interactions with necroptosis pathways remain to be fully elucidated. Future studies incorporating multi-center clinical samples and mechanistic experiments will be essential to validate and extend these findings.

## 5 Conclusion

Identification of necroptosis-related lncRNAs provides valuable insight into prognosis prediction and personalized management of PCa, with potential benefits for both survival and quality of life. Targeting necroptosis and lncRNAs may represent a promising strategy to overcome resistance to current systemic therapies and enhance the efficacy of immunotherapy. Future studies will further elucidate their biological roles and therapeutic potential. Finally, these efforts may enable the translation of necroptosis-related lncRNA signatures into precise and effective clinical interventions for PCa.

## Data Availability

The original contributions presented in the study are included in the article/supplementary material, further inquiries can be directed to the corresponding author.
